# RpbL12 Assists Catalysis by Correctly Positioning the Incoming Aminoacyl-tRNA in the A-Site of *E. coli* 70S Ribosomes

**DOI:** 10.2174/1874091X01812010113

**Published:** 2018-07-31

**Authors:** Jean-Bernard Créchet, Fulbert K. Agbo’Saga, Soria Baouz, Codjo Hountondj

**Affiliations:** 1 Ecole Polytechnique, Route de Saclay, F-91120 Palaiseau, France; 2 Sorbonne Université, Campus Pierre et Marie Curie, Unité de Recherche SUUR6 “Enzymologie de l’ARN”, 7 Quai Saint-Bernard, F-75252 Paris Cedex 05, France

**Keywords:** *E. coli* ribosomal protein bL12, GGQ-like GAN motif of bL12, Lys-65 of bL12, Site-directed mutagenesis, Mechanism of peptide bond formation, Aminoacyl-tRNA

## Abstract

**Introduction:**

We have recently demonstrated that Lys-65 of the 62GANK65 motif of *E. coli* RpbL12 was affinity labeled with a tRNA analogue, resulting in the loss of activity.

**Materials and Methods:**

In this report, we show that mutations operated at the position of Lys-65 led to an impairment in the activity of the mutant ribosomes, except the K65R or K65H bL12 mutants, suggesting that the only requirement of the reaction catalyzed or facilitated by RpbL12is the positive charge of the side chain of Lys-65. We also demonstrate that Lys-65 did not play any role in the peptidyl transferase reaction with respect to puromycin, but rather assisted the binding of the incoming aminoacyl-tRNA to the ribosomal A-site.

**Results & Discussions:**

The protonated, positively charged εNH_3_^+^ form of Lys-65 is likely to participate to the binding of aa-tRNA through ionic bonds with phosphate groups, in order to insure the accurate positioning required for the nucleophilic attack of its α-amino group on the carbonyl carbone of peptidyl-tRNA.

**Conclusion:**

This α-NH_2_ group is likely to be generated by the unprotonated εNH_2_ form of Lys-65 which is capable of withdrawing a proton from the α-NH_3_^+^ group of aa-tRNA.

## 1 INTRODUCTION

Ribosomes are the largest ribonucleoproteic particles in living cells. They consist of a large and a small subunits having each separate functions. The large ribosomal subunit (50S in eubacteria and archaeabacteria, and 60S in eukaryotes) contains the Peptidyl Transferase Center (PTC), the catalytic site where peptide bond formation occurs, while the small subunit (30S in bacteria and 40S in eukaryotes) contains the site where mRNA sequences are decoded. The current view of the PTC is that peptide bond formation is catalyzed exclusively by ribosomal RNA (rRNA), and that consequently, the ribosome is a ribozyme [1, 2]. However, in sharp contrast with the ribozyme-catalyzed peptidyl transfer mechanism, there is increasing evidence that the eukaryote-specific large subunit ribosomal protein eL42 could contribute to the catalytic activity of the ribosomes at the elongation step of translation [3-5]. This protein is strongly conserved in eukaryotes and archaea, and had been previously shown to make part of the PTC [6]. In fact, by using an affinity labeling strategy, Fabijanski and Pellegrini [6] demonstrated 36 years ago that rp eL42 is a major protein component of the PTC of rat liver ribosomes. To this end, they had designed a 3’-terminal pentanucleotide fragment C-A-C-C-A(Acetyl[^3^H]Leu) in which mercury atoms ([^203^Hg]) had been attached to the C-5 position of all three cytosine residues, for affinity labeling rat liver 80S ribosomes [6]. This mercurated fragment could covalently bind to a small number of rat large subunit ribosomal proteins, eL42 being the major labeled rp [6]. Therefore, eL42 (formerly L36a) was considered as a protein component of the PTC of eukaryotic 80S ribosomes in the protein database (PDB Accession Number P83881). Later on, eL42 was shown to contact the CCA-end of P-site bound tRNA on human 80S ribosomes [3], suggesting that this large subunit ribosomal protein plays a functional role at the PTC. Recently, the CCA-end of a tRNA in the P/P state was shown to interact in majority with a lysyl residue (Lys-197) in the neighborhood of the GGQ motif of eRF1 bound to an A-site stop codon [7]. This result suggests that the CCA-arm of P-tRNA binds to the GGQ motif of eRF1, as previously predicted by crystallographic data [8]. In addition, this interaction can be considered as functionally important, since it is supposed to result in the hydrolysis of P-site bound peptidyl-tRNA, at the termination of translation [8-12]. We have also demonstrated that Lys-53 of the 49GGQTKP54 motif of eL42 binds to the CCA-end of a tRNA at the P/E hybrid site on the human 80S ribosomes [4], suggesting that, similarly to the GGQ motif of A-site bound eRF1, the GGQ motif of eL42 is likely to play a functional role at the PTC. More recently, we have demonstrated that the CCA-end of P-site bound tRNA contacts both the GGQ regions of eL42 and of an A-site bound translation termination factor eRF1, in accordance with the observation by numerous research groups that the GGQ motif triggering the hydrolysis of peptidyl-tRNA should contact the PTC of the ribosome [8-14]. Finally, the proposition that eL42 would represent the PTC whose interaction with the GGQ motif of eRF1 could result in the hydrolysis of peptidyl-tRNA was recently strengthened by the demonstration that the monomethylated Gln-51 and Lys-53 residues contained in the 47GFGGQTK53 sequence of eL42 and the monomethylated GGQ motif of eRF1 represents the sites of interaction between these two proteins through hydrophobic contacts between methyl groups on the human 80S ribosomes [15]. Similarly, it was recently demonstrated that the large subunit ribosomal protein bL12 contacts the CCA-end of P-site bound tRNA on *E. coli* 70S ribosomes [16]. In fact, Lys-65 residue of the 62GANK65 motif of bL12 which was shown to be affinity labeled with a reactive tRNA analogue, matches with Lys-53 of the 49GGQTK53 motif of eL42 [16] which was found labeled with the same reactive tRNA analogue in a previous study [4]. These results are consistent with a functional role for both residues at the tRNA-CCA binding site of these proteins on the ribosomes. Moreover, the GANK and GGQTK motifs occupy respectively comparable positions in the 3-D structures of these proteins [16]. In the 3-D structure of human eL42 modeled by homology with the archaeal counterpart from *Haloarcula marismortui*, the GGQTK peptide is located in an extended loop at one extremity [5, 16]. Similarly, the GANK peptide is located at the tip of the crystallographic structure of the C-terminal domain of bL12 [16-19]. The possibility that rp bL12 could contribute to the catalytic activity of the *E. coli* 70S ribosomes is the subject of this study. Mutational analysis of the GGQ-like GANK motif of *E. coli* bL12 has shown that amino acid changes in this conserved region result in reduced poly(U)-dependent poly(Phe) synthesis activity of the 70S ribosomes. It should be recalled that bL12 and eL42 are respectively the new names for the eubacterial large subunit ribosomal protein L7/L12 and the eukaryal or archaeal ribosomal proteins of the L44E family (formerly L42A or L42AB in yeast or L36a in human, or L44e in archaea) [20]. Accordingly, bL12 currently refers at the same time to the eubacterial protein L12 with a free NH_2_ terminus and to the NH_2_-terminally acetylated L7 chain whose amino acid sequence is identical to that of L12. However, the present study describes the activities of ribosomes reconstituted respectively with L12 and L7, the latter protein being eventually referred to as bL7 [20]. Therefore, for clarity, when necessary, we will preferably use the ancient system for naming ribosomal proteins, in order to distinguish between these two almost identical proteins.

## 2 MATERIALS AND METHODS

### 2.1 Materials

Poly(U), puromycin and tRNA^Phe^ from *E. coli* were from Sigma-Aldrich and L-[^14^C(U)]Phenylalanine (18 GBq/mmol) or [^3^H]Phenylalanine (4740 GBq/mmol) from Perkin Elmer. tRNA^Phe^ was aminoacylated using [^14^C(U)]Phenylalanine or [^3^H]Phenylalanine with an excess amount of partially purified phenylalanyl-tRNA synthetase from *E. coli* [21]. [^3^H]Phe-tRNA^Phe^ (128 GBq/mol) was acetylated following the method described by Haenni and Chapeville [22].

### 2.2 Methods

#### 2.2.1 Purification of the Recombinant E. coli Wild-type or Mutant bL12 Proteins

Wild-type or mutated his-tagged proteins were expressed in *E. coli* BL21(DE3) strain transformed with recombinant plasmid pET21L12C-6His (a generous gift of Dr. S. Sanyal) or with mutagenized pET21L12C-6His. Cells were grown at 37°C in 1 liter of LB rich medium containing 50 µg.ml^-1^ ampicillin to 0.5 A_600_, after which induction with 0.2 mM isopropyl-ß-D-thiogalacto-pyranoside took place at 21°C with incubation up to 20 hours. After harvest, the cells were washed in PBS, sonicated 15 times for 10 s at 4°C in 30 ml buffer A (25 mM Tris-HCl pH 7.5, 0.5 M NaCl, 1 mM ß-ME, 10 mM Imidazole, 0.05% Tween 20, 1 mM MgCl_2_ containing 0.5 mg.ml^-1^ lysosyme, 100 µg.ml^-1^ DNase, one tablet of complete mini EDTA free protease inhibitor cocktail (Roche Diagnostics). The supernatant obtained by centrifugation (100,000 X g for 30 minutes) was applied two times on 1 ml His GraviTrap affinity column (GE Healthcare) equilibrated with buffer B (25 mM Tris-HCl pH 7.5, 0.5 M NaCl, 1 mM ß-ME, 10 mM Imidazole), the column was washed successively with buffer B containing 50 mM NaCl and 100 mM imidazole before elution of the protein with 8 ml of the same buffer containing 0.4 M imidazole. This fraction was concentrated by ultrafiltration, dialysed against buffer C (50 mM Tris-HCl pH 7.5, 0.3 M NaCl, 7 mM ß-ME, 50% glycerol) and stored at -25°C.

#### 2.2.2 Purification of the Acetyl-Transferase RIML

The *RIML* gene of *Escherichia coli* encodes an enzyme catalyzing the acetylation of the N-terminal serine of ribosomal protein L12, thereby converting it into L7 [23]. *RIML* gene was amplified using Pwo polymerase high fidelity PCR system (Roche Diagnostics) from *E. coli* MRE600 genomic DNA isolated following the instructions of E.Z.N.A bacterial DNA kit from OMEGA-Bio-tek and the 5’ primer CACCATGACTGAAACGATAAAAGTAAGC and the 3’ primer TTATTGTGAATCGATAATACGCGCG including the 5’ and 3’ extremities of the gene. The amplified fragment was purified and cloned into pET151/D-TOPO following the TOPO cloning procedure (Invitrogen). This vector allows expression of recombinant protein with an N-terminus containing the V5 epitope, a 6XHis tag and a TEV protease cleavage site. Accuracy of the cloning was analyzed by sequencing. BL21(DE3) cells were transformed with pET151/D-TOPO/RIML plasmid. Cells were grown at 37°C in 2 liters of LB rich medium containing 50 µg.ml^-1^ ampicillin to 0.5 A_600_, after which induction with 0.1 mM isopropyl-ß -D-thiogalacto-pyranoside took place at 20°C with incubation overnight. After harvest, the cells were washed in PBS, sonicated 15 times for 10 s at 4°C in 40 ml buffer A (25 mM Tris-HCl pH 7.5, 0.5 M NaCl, 1 mM ß-ME, 10 mM Imidazole, 0.05% Tween 20, 10% glycerol, 1 mM MgCl_2_ containing 0.5 mg.ml^-1^ lysosyme, 100 µg.ml^-1^ DNase, one tablet of complete mini EDTA free protease inhibitor cocktail (Roche Diagnostics). The supernatant obtained by centrifugation (100, 000 X g for 30 minutes) was applied two times on 2 ml Ni-NTA superflow column (Qiagen) equilibrated with buffer B (25 mM Tris-HCl pH 7.5, 0.5 M NaCl, 1 mM ß-ME, 10 mM Imidazole), the column was washed with buffer B containing susccessively 10 and 20 mM imidazole before elution of the protein with 8 ml of the same buffer containing 0.4 M imidazole. This fraction (about 110 mg) was concentrated by ultrafiltration, dialysed against buffer C (25 mM Tris-HCl pH 7.5, 1 mM MgCl_2_, 5 mM ß-ME, 50% glycerol) and stored at -25°C. 10 mg of RIML acetyl-transferase was treated with 1000 units of 6XHis tag AcTEV protease to cleave the double 6XHis/V5 epitope tag in 50 mM Tris-HCl pH 8, 0.5 mM EDTA, 0.3 mM DTT) for 20 hours at 10°C.

The mixture, after addition of 0.5 mM MgCl_2_ and 10 mM imidazole was loaded on Ni-NTA superflow equilibrated with buffer A and the acetyl tansferase protein was isolated free of the double tag and the Ac-TEV protease which were retained on the resin.

#### 2.2.3 *In Vitro* Acetylation of the Recombinant *E. coli* L12 Protein

180 µM L12 were incubated in 5ml 10 mM Tris-HCl pH 7.5, 1 mM MgCl_2_, 1mM DTT in the presence of 12 µM purified RIML acetyl-transferase and 0.6 mM of N-acetyl coenzyme A (N-acetyl-CoA or N-Ac-CoA) for 1 hour at 30°C and overnight at 4°C. Conditions of acetylation were preliminarily determined in the presence of increasing concentrations of *RIML* product and [^3^H]-N-Ac-CoA (specific activity, 2.7 Bq.pmol^-1^). The amount of [^3^H]-N-acetylated protein L12 was then determined by filtration on nitrocellulose discs that were washed twice with 3ml of ice-cold 10 mM Tris-HCl pH 7.5, 1 mM MgCl_2_, 1mM DTT buffer and then counted for radioactivity.

#### 2.2.4 Purification of *In Vitro* Acetylated Rp L12

5 mM Imidazole and 0.3 M NaCl were added to the 5 ml L12 acetylation reaction mixture then loaded on 1 ml Ni-NTA superflow column (Qiagen) equilibrated with buffer B in order to eliminate the *RIML* acetyl-transferase and the excess of acetyl-coA which were eluted in the washing buffer B before the elution of 6His-L7 protein in the same buffer containing 0.4 M imidazole. After concentration by ultrafiltration and dialysis against buffer C, the L7 preparation was stored at -25°C.

#### 2.2.5 MALDI Mass Spectrometric Analyses of the Acetylated L7 or Non Acetylated L12 Forms of BL12

After elution from the Superdex column, the fractions of interest were pooled and solutions of the NH_2_-terminally acetylated (L7) or non acetylated (L12) forms of bL12 were each desalted using a Ziptip C4 (Millipore Saint Quentin en Yvelines France), and the proteins were eluted with 10 µl of a solution of 39% water 1% formic acid and 60% acetonitrile and analyzed by MALDI mass spectrometry. Samples (1 μl) were loaded on a MALDI plate using a solution of 5 mg/ml of α-Cyano-4-hydroxycinnamic acid in 30% (water 0.1% TFA) 70% acetonitrile. Mass spectra were obtained on an Autoflex speed series MALDI-TOF mass spectrometer (Bruker Daltonics) equipped with a pulsed N2 laser (337 nm) in a positive reflectron mode. Ions formed by the laser beam were accelerated to 23 keV. The final spectra were obtained by accumulating ~1500 single laser shots.

#### 2.2.6 Site-directed Mutagenesis

Mutations were introduced with the QuickChange II XL Site-Directed Mutagenesis Kit and by using the manufacturer (Stratagen) recommended protocol. As a substrate for the mutagenesis reactions we used pET21L12C-6His plasmid. The mutagenic oligonucleotides containing the specific modified bases and their respective complementary primers for introducing the required amino acid substitutions on bL12 were synthesized and purified by Eurogentec. Following transformation of competent XL10-Gold cells, minipreps on selected transformants were analyzed by sequencing to verify the presence of desired mutations and to check the absence of secondary mutations.

#### 2.2.7 Preparation of Elongation Factors and Ribosome Subunits

*E. coli* elongation factors, EF-Tu, EF-Ts and EF-G were purified as described in [16]. *E. coli* 70S ribosomes were prepared as reported in Sander *et al*. [24]. Ribosomal 30S and 50S subunits were separated and purified by centrifugation in a Beckman SW32Ti rotor through a 15-30% sucrose gradient in 50 mM Tris-HCl pH 7.5, 30 mM KCl, 30 mM NH_4_Cl, 0.5 mM MgCl_2._ The sucrose gradients were formed using the Biocomp gradient master and collected after 16 hours centrifugation (30,000 X g) with a Biocomp piston gradient fractionator. Ribosomal particles were then concentrated by centrifugation for 5 hours (150,000 X g) in a Beckman 60Ti rotor, resuspended in ribosome buffer: 50 mM Tris-HCl pH 7.5, 30 mM KCl, 30 mM NH_4_Cl, 10 mM MgCl_2,_ 7 mM ß-ME and stored at -25°C in the same buffer containing 50% glycerol. The large 50S ribosomal subunit deprived from the bL12 (L7/L12) protein is referred to as 50SΔ in the present report. It was obtained as follows: the protein was extracted at 4°C from 50S subunit in ribosome buffer brought gradually to 1M NH_4_Cl and 20 mM MgCl_2._ in 7 ml final volume. Half volume of precool absolute ethanol was then added. After 10 minutes treatment, the solution was spun at 25,000 X g for 10 minutes at -10°C. The pellet resuspended in 1 ml of ribosome buffer was treated again in the same way with 1M NH_4_Cl and 33% ethanol. The combined supernatants contain bL12 (L7/L12). The pellet containing 50S particles depleted of bL12 was suspended, dialysed in ribosome buffer with 50% glycerol and stored at -25°C.

#### 2.2.8 Poly(Phe) Synthesis Activity of *E. coli* 70S Ribosomes

Poly(Phe) synthesis was determined as incorporation of L-[^14^C(U)]Phenylalanine into hot trichloroacetic acid-insoluble material, as previously described [25]. The reaction mixture contained 40 mM Tris-HCl pH 7.5, 7 mM MgCl_2_, 80 mM NH_4_Cl, 1 mM dithiothreitol, 1 mM ATP, 1 mM Phosphoenolpyruvate, 0.5 mM GTP, 50 µg.ml^-1^ pyruvate kinase, 5 µM tRNA^Phe^ (first charged during a 30 min incubation at 30°C with a 2 fold excess of L-[^14^C(U)]Phenylalanine (5 GBq.mmol^-1^)) and a saturating amount of partially purified phenylalanine synthetase), 3.5 µg poly(U) and 0.3 µM EF-Tu, 0.3 µM EF-Ts, 0.2 µM EF-G and the indicated amount of 30S, 50S ribosomal subunits and bL12. After incubation, aliquots were withdrawn spotted on glass fiber filters and hot trichoroacetic acid-insoluble radioactivity was determined.

#### 2.2.9 Peptidyl Transferase Activity

Reconstitution of ribosome particles was performed during incubation at 37°C for 10 min in 85 µl buffer A (50 mM Tris-HCl pH 7.5, 30 mM KCl, 30 mM NH_4_Cl, 10 mM MgCl_2,_ 1mM DTT) containing 30S (1.4 µM), untreated 50S (1.4 µM) or 50S depleted of bL12 (1.4 µM) in the absence or presence of 10 µM purified wild type bL12 or mutated bL12s. 3 µM Ac-[^3^H]Phe-tRNA^Phe^ (128GBq/mol), 20 µg poly(U), were then added to the reconstituted particles to form “Ac-[^3^H]Phe-tRNA^Phe^.poly(U).ribosome” complexes during incubation for 15 min at 30°C in a total volume of 100 µl. These complexes were then separated on superose 12 HR 10/30 column (GE Healthcare) equilibrated in the same buffer. The isolated complexes were concentrated to 200 µl using microcon 100 from Amicon and the concentration of the complexes was determined (one A_260_ unit of “Ac-aa-tRNA.mRNA.ribosome” complex was assumed to contain 24 pmol of ribosome complex).

Peptidyl transferase activity was determined in 50 µl reaction mixture containing in buffer A, 0.3 µM of “Ac-[^3^H]Phe-tRNA^Phe^.poly(U).reconstituted ribosome” complex, with or without 0.5 µM EF-G and 0.5 mM GTP, in the presence or absence of 1.5 mM Puromycin. After 15 min incubation at 30°C, 47 µl were withdrawn and placed on Whatman GFC filters. The filters were washed with the cold trichloroacetic acid procedure, dried and then assayed for radioactivity. The extent of puromycin reactivity (PTC activity) was determined by comparing the radioactivity of the unreacted acid-insoluble material in the presence and in the absence of the antibiotic. 100% activity corresponds to the binding of Ac-[^3^H]Phe-tRNA^Phe^ to the ribosomal sites determined in the absence of puromycin.

#### 2.2.10 Enzymatic Binding of [^3^H]Phe-tRNA^Phe^ to the Ribosomal A-Site

The enzymatic binding of [^3^H]Phe-tRNA^Phe^ to the ribosomal A-site was performed as previously described [26]. Reconstitution of ribosome particles was first performed during incubation at 37°C for 10 min in 20 µl buffer A containing 1.6 µM 30S subunits, 1.6 µM 50S subunits untreated or depleted of rp bL12, in the absence or presence of 12 µM purified wild-type or mutant rps bL12. Poly(U) (14 µg) and tRNA^Phe^ (1 µM) were then added and the incubation was prolonged for 15 min at 30°C in a 33 µl mixture. Preformed EF-Tu•GTP complex was prepared by incubating for 10 min at 30°C 3.3 µM EF-Tu•GDP in 25 mM Tris-HCl pH 7.5, 5 mM EDTA, 1 mM DTT with 0.5 mM GTP and then stabilized by the addition of 6 mM MgCl_2_. 15 µl containing 40 mM Tris-HCl pH 7.5, 7 mM MgCl_2_, 80 mM NH_4_Cl, 1 mM dithiothreitol, 0.5 mM GTP, 50 µg.ml^-1^ pyruvate kinase 0.4 µM [^3^H]PhetRNA^Phe^ (specific activity: 128 GBq/mmol) were preincubated for 10 min at 30°C in the presence or absence of 0.4 µM preformed EF-Tu•GTP before reaction was started with the addition of 15 µl of poly(U) programmed reconstituted ribosomes with final concentrations of 0.4 µM 30S and 50S, 5 µM purified wild type bL12 or mutated bL12s, 0.4 µM tRNA^Phe^ and 7 µg poly(U). The enzymatic binding mediated by EF-Tu•GTP and non enzymatic binding of [^3^H]Phe-tRNA^Phe^ to the ribosomal A site was determined after 8 min at 30°C by the nitrocellulose binding assay.

## 3 RESULTS AND DISCUSSION

### 3.1 Tertiary and Quaternary Structures of the *E. coli* Ribosomal Endogenous or Recombinant bL12 Protein

The so called eubacterial ribosomal “stalk” protein L7/L12 was previously described either as an NH_2_-terminally acetylated form (L7 also called bL7), or as a polypeptide with a free NH_2_ terminus, L12, both forms of the protein being currently named bL12 in the new nomenclature of the ribosomal proteins [20]. Cryo-electron Microscopy [27] studies had permitted to visualize the L7/L12 protein in the *E. coli* 70S ribosome as two dimers (L7/L12)_2_ (or [bL7/bL12]_2_) bound to one copy of protein L10 (or uL10), which anchors the pentamer to the large 50S ribosomal subunit [27]. The C-Terminal Domain (CTD) of protein L7/L12 was the first ribosomal protein to be studied by X-ray crystallography [17, 18]. Each monomer is built up with two distinct domains linked by a flexible hinge (residues 37-52): an elongated N-terminal domain (residues 1-36) that represents the site of dimerization and of the binding to L10, and a large globular C-terminal domain (residues 53-120) containing the site of interaction with the elongation factors [28, 29]. It is interesting to note that the tertiary and quaternary structures of rp L7/L12 (bL12), as deduced from various structural studies in solution and in the ribosome, or from the crystal structure of the whole protein are comparable in solution and in the ribosome [30]. These results had been interpreted to reflect the independence of the 3-D structure of the pentameric (L7/L12)_2_-L10 complex with regard to the whole 50S ribosomal subunit [30]. Therefore, it is most probable that the recombinant *E. coli* rp L12 presents the same tertiary and quaternary structures as the endogenous protein. To address the question of the quaternary structure of the recombinant L12 protein used in the present study, we have analyzed this protein by chromatography on a Superdex 75 column. The His-tagged rp bL12 eluted from the His GraviTrap affinity column was applied onto the Superdex 75 column. Elution was monitored by the absorbances at 280 nm and 260 nm (Fig. **1A**). Calibration of the column with protein markers indicated an apparent molecular weight of about 50 kDa for the recombinant *E. coli* rp L12, suggesting that this protein presents the associated form (L7/L12)_2_ corresponding to the assembly of 4 monomers of 13 kDa, as previously reported for the native endogenous *E. coli* rp L7/L12 [31]. Such association of L12 polypeptide chains is likely to reflect a self-assembly property specific to rp L7/L12 [31].

### 3.2 *In Vitro* Acetylation of bL12

The fact that the NH_2_-terminally acetylated domain (residues 1-36) is responsible for dimer interaction [17, 18] prompted us to check the role of acetyl groups in the dimerization process. To this end, we have prepared a fully acetylated (L7)_4_ [or (bL7)_4_] sample where the free NH_2_-terminus of the two L12 chains of rp (L7/L12)_2_ have been acetylated in the presence of the enzyme RIML acetyl-transferase from *E. coli* and of N-acetyl coenzyme A (N-Ac-CoA) as donor of acetyl groups (Materials and Methods). Analysis by chromatography on the same Superdex 75 column of the fully acetylated 6His-L7 protein eluted from the Ni-NTA superflow column (Materials and Methods) resulted in an elution profile identical to that of L12 (Fig. **1A**). When the fully acetylated 6His-L7 protein was obtained by incubating the 50 kDa (L7/L12)_2_ protein eluted from the Superdex column with the incubation mixture of the acetylation reaction, followed by application of this mixture to the same Superdex column, the same elution profile was observed (molecular weight of 50 kDa for (L7)_4_) (Fig. **1A**). These results suggest that acetylation of all four polypeptide chains in the (L7)_4_ assembly did not give rise to a different association mode for this multimeric rp. Finally, it was verified by an SDS-PAGE analysis that the (L7/L12)_2_ or (L7)_4_ samples to be used in the present study are homogenous (Fig. **1B**). In addition, Fig. (**1B**) shows that both (L7/L12)_2_ and (L7)_4_ assemblies are made of the same polypeptide chain of about 13 kDa, as expected. We next asked whether the acetylation of all polypeptide chains in rp (L7)_4_ has any effect on the activity of 70S ribosomes reconstituted with this protein. We addressed this question by comparing the poly(U)-dependent poly(Phe) synthesis activities of *E. coli* 70S ribosomes reconstituted with rps (L7/L12)_2_ or (L7)_4_. However, a prerequisite for this study is the demonstration that all four polypeptide chains of rp (L7)_4_ are effectively acetylated. It should be recalled that a molecular mass of 13228 Da had been calculated for the polypeptide chain of the recombinant *E. coli* L12 protein used in this study, while the measured molecular mass, as determined by ESI mass spectrometry had been found equal to 13228 Da [16]. Interestingly, in the MALDI mass spectrometric analysis of the fully acetylated (L7)_4_ sample presented here, the peak of mass 13274 Da in Fig. (**2B**) showed a mass value increased by 42 Da (the molecular mass of an acetyl group) relative to the calculated mass of 13228 Da and a mass deviation of only 4 Da. This result, together with the absence of a 13228 Da peak in Fig. (**2B**) indicate that only the acetylated polypeptide chain is present in the sample of (L7)_4_. The presence of a very low 13499 Da peak in Fig. (**2B**) might be explained by the fact that, in addition to the acetylation of the L12 chain at its N-terminus, the enzyme RIML acetyl-transferase has attached acetyl groups to other acetylatable amino acid residues of the protein such as lysyl residues. For example, the peak of mass 13499 Da showed a mass value increased by 225 Da relative to the mass of 13274 Da corresponding to the N-terminally acetylated polypeptide chain (L7). The difference of 225 Da is likely to correspond to the attachment of five acetyl groups (total 210 Da) and one methyl group (15 Da). As a control, the molecular weights measured with the sample of (L7/L12)_2_ are 13228 Da and 13270 Da (Fig. **2A**). The difference between the two masses (42 Da) correlates with the acetylation of a polypeptide chain of each (L7/L12) dimer, as expected. The two very low peaks at 13285 Da and 13327 Da correspond respectively to the acetylated L7 chain (MW 13270 Da) modified with a methyl group (MW 15 Da) or with both a methyl group and a supplementary acetyl group (42 Da) (total mass increase of 57 Da) (Fig. **2A**).

### 3.3 Poly(U)-Dependent Poly (Phe) Synthesis Activity of *E. coli* 70S Ribosomes Reconstituted with Rps (L7/L12)_2_ or (L7)_4_

**Fig. (3):**
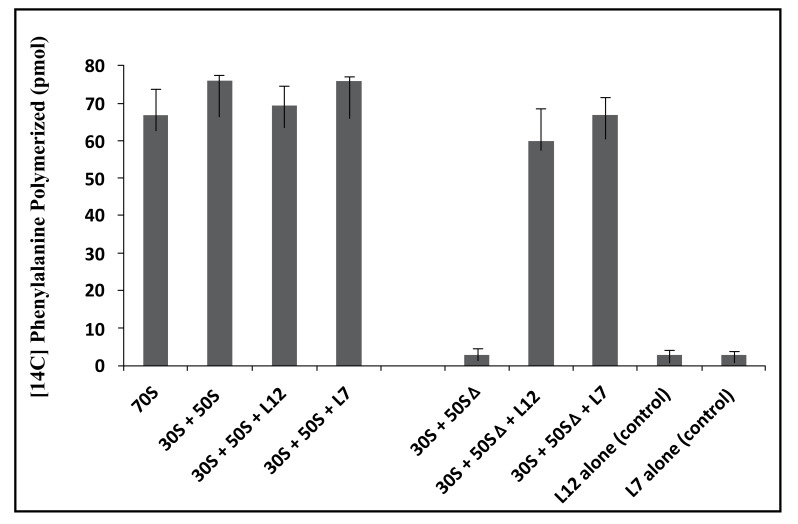
**Poly(U)-dependent poly(Phe) synthesis activity of *E. coli* 70S ribosomes reconstituted with rps (L7/L12)_2_ or (L7)_4_.** Purified *E. coli* 30S subunits (0.65 µM) and purified 50S subunits (0.65 µM) untreated or depleted from rp bL12 were incubated for 10 min at 37°C in the presence of 10 µM purified rps (L7/L12)_2_ or (L7)_4_ in 15 µl of 50 mM Tris-HCl pH 7.5, 30 mM KCl, 30 mM NH_4_Cl, 10 mM MgCl_2,_ 1mM DTT, before adding all the components required for poly(Phe) synthesis, as described in Materials and Methods. The activity (expressed as the amount (pmol) of poly-[^14^C]Phe synthesized) was determined after 12 min incubation at 30°C in 35µl total reaction mixture.

In this study, the poly(U)-dependent poly(Phe) synthesis activity was measured on two types of 70S ribosomes: the first type called wild-type was obtained by the combination of equimolar amounts of the *E. coli* small (30S) and large (50S) ribosomal subunits. The second, called reconstituted wild-type is the 70S ribosome whose large subunit was reconstituted by adding the recombinant wild-type rp bL12 to 50S subunits which had been depleted from the original rp L7/L12 copy (namely 50SΔ ribosomal subunits) prior to the addition of the recombinant wild-type protein. In fact, it was previously demonstrated that rp bL12 can be removed easily and selectively from the large ribosomal subunit, leading to a severe reduction of the rate of protein synthesis [32]. Thus, the original rp L7/L12 copy can be replaced by variant bL12 proteins. Taking advantage of this special feature, Sanyal’s group in Uppsala (Sweden) has constructed a novel *E. coli* strain JE 28, in which a (His)6-tag has been inserted at the C-terminus of bL12 [32]. The latter His-tagged recombinant rp bL12 is the one that we used in this work. As shown in Fig. (**3**), depletion of the endogenous rp L7/L12 from the 50S subunit abolished the poly(U)-dependent poly(Phe) synthesis activity of the wild-type 70S ribosomes, in agreement with previous studies [32], while further addition of the recombinant wild-type L12 protein fully restored the activity (Fig. **3**). This result suggests that rp L12 participates to the activity of the 70S ribosomes, as previously reported [32]. In addition, the activity of the *E. coli* 70S ribosomes reconstituted with the fully acetylated (L7)_4_ protein were found to be comparable with that of the ribosomes reconstituted with rp (L7/L12)_2_ (Fig. **3**), suggesting that the acetylation did not significantly affect the activity. The control experiments with rp (L7/L12)_2_ or rp (L7)_4_ show that these proteins alone were not capable of catalyzing poly(Phe) synthesis (Fig. **3**). These results suggest that the functional role of rp L7/L12 is linked with its presence into the 70S ribosome.

### 3.4 Amino Acid changes in the 62GANK65 Peptide of bL12 Result in Reduced Poly(U)-Dependent Poly(Phe) Synthesis Activity of Reconstituted *E. coli* 70S Ribosomes

**Fig. (4):**
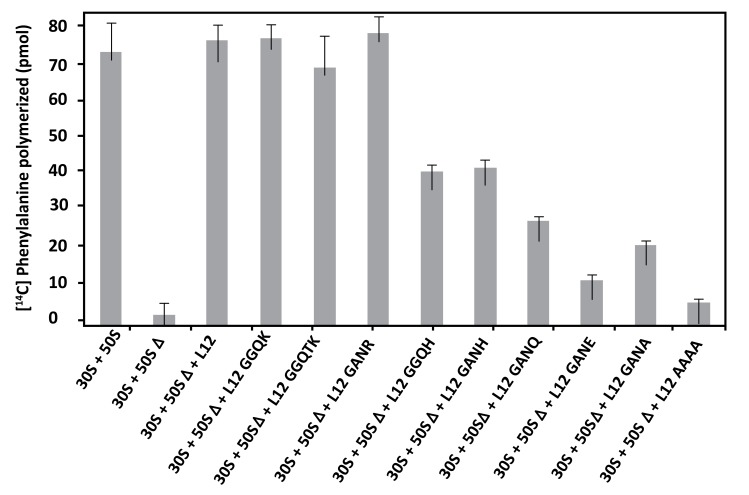
**Poly(U)-dependent poly(Phe) synthesis activity of *E. coli* 70S ribosomes reconstituted with mutant rps (L7/L12)_2_.** Purified *E. coli* 30S subunits (0.65 µM) and purified 50S subunits (0.65 µM) untreated or depleted from rp bL12 were incubated for 10 min at 37°C in the presence of 10 µM purified wild type or mutated rps bL12. The experimental conditions were the same as in Fig. (**3**).

The *E. coli* large subunit ribosomal protein bL12 has been recently shown to be evolutionarily conserved with the eukaryote-specific rp eL42 at the tRNA-CCA binding site of the ribosomes [16]. These two 12 kDa proteins have been shown to display primary structure similarities, with an average of 40-50% of identities and conservative replacements [16]. In particular, the 62GANK65 motif of bL12, the Lys-65 residue of which had been recently shown to be affinity labeled with a reactive tRNA analogue, matches with the 49GGQTK53 motif of eL42 [16]. Lys-53 of this motif was found labeled with the same reactive tRNA analogue in a previous study [4]. It has been also recently demonstrated that Lys-65 of *E. coli* bL12 and Lys-53 of human eL42 exhibit an abnormally low pK, as compared with the one that is normally found for the side chain of a lysine residue (pK 10.5) [5, 16]. Finally, Lys-55 of *S. pombe* (Lys-53 in human) rp eL42 was shown to contribute to the catalytic activity of the yeast ribosome at the elongation step of translation (C. Hountondji, personal communication). To address the question of the functional role of the GGQ-like GANK motif of *E. coli* rp bL12, and to evaluate its contribution to the activity of 70S ribosomes, we compared the activities of the *E. coli* wild-type and mutant ribosomes in the poly(U)-directed poly(Phe) synthesis activity. To this end, we have constructed different mutants of *E. coli* bL12 with the help of site-directed mutagenesis by changing one or several residues in this motif. The bL12 mutants obtained were used to reconstitute the *E. coli* 70S ribosomes from a mixture of intact small (30S) subunits and of the aforementioned large 50SΔ subunits. As shown in Fig. (**4**), the poly(U)-dependent poly(Phe) synthesis activity of the wild-type or the reconstituted wild-type 70S ribosomes, as well as that of the mutant ribosome reconstituted with the K65R mutant rp bL12 protein were of the same order of magnitude. By contrast, the activity of the mutant 70S ribosomes reconstituted with other mutant rp bL12 proteins was gradually decreased in the order K65H>K65Q>K65A>K65E. On one hand, the decrease of activity in all mutant ribosomes except the one comprising the K65R mutant bL12 (Fig. **4**) suggests that all the mutations operated at the position of Lys-65 of the 62GANK65 motif of bL12 led to the impairment in the poly(U)-dependent poly(Phe) synthesis activity. This result suggests that the Lys-65 residue of bL12 is critical for the activity of *E. coli* 70S ribosomes. On the other hand, the fact that the wild-type or the mutant ribosomes reconstituted with rp bL12 mutants with K65R or K65H substitutions showed the highest activity with regard to the other mutant ribosomes suggests that the only requirement of the reaction catalyzed or facilitated by rp bL12 is the positive charge of the side chain of the amino acid residue at this position. For example, when Lys-65 was changed to His-65, the poly(U)-dependent poly(Phe) synthesis activity, as compared with that of the wild-type 70S ribosomes was decreased by 45% on average (Fig. **4**), in accordance with the fact that His is less positively charged than Lys at neutral pH. By contrast, the activity of the mutant ribosome reconstituted with the K65R mutant rp bL12 protein was slightly superior to that of the wild-type or the reconstituted wild-type 70S ribosomes. This result is consistent with Arg being the most positively charged amino acid residue. The activity of the ribosomes reconstituted with rp bL12 mutants with the K65A or K65Q substitutions is comparably reduced (Fig. **4**), while that of the ribosomes comprising the K65E substitution is more severely reduced. It should be noted that glutamine and glutamic acid are polar amino acid residues the side chains of which are much larger than that of Ala and comparable in size with that of lysine and containing a neutral or a negatively charged side chain, respectively. Therefore, one possible interpretation of these results is that, whatever the size of the side chain of the amino acid residue introduced at position 65, the absence of a positive charge is less dramatic than the presence of a negative charge. At this stage one can anticipate that the role of the positively charged Lys-65 side chain might imply the interaction with a negative charge of a protein or RNA component on the ribosome. It is interesting to note that introduction of three mutations (G62A/N64A/K65A) resulting in the replacement of the GANK peptide by AAAA led to a more pronounced reduction in the activity (Fig. **4**). This result would suggest that other residues of the GANK motif are important for the activity of the ribosomes. For example, it is most probable that the Gly residue is involved in the formation of the GANK loop [16], similarly to the glycines of the GGQ loop of eRF1 that had been shown to play a structural role essential for the hydrolysis of the peptidyl-tRNA at the termination of translation [8-12]. Finally, when the GANK peptide was replaced by GGQK or GGQTK, the *E. coli* 70S ribosomes reconstituted with the rps bL12 obtained were found to be fully active in poly(U)-dependent poly(Phe) synthesis, suggesting that the GANK loop of bL12 and the GGQ loop of eL42 are similarly folded as discussed in a previous report [16]. A control experiment consisting in measuring the poly(Phe) synthesis activity of rp L7/L12 showed that this protein alone was not capable of catalyzing poly(Phe) synthesis, as already demonstrated in Fig. (**3**).

### 3.5 Amino Acid Changes in the 62GANK65 Peptide of bL12 did not Affect the Peptidyl Transferase Activity of *E. coli* 70S Mutant Ribosomes

**Fig. (5):**
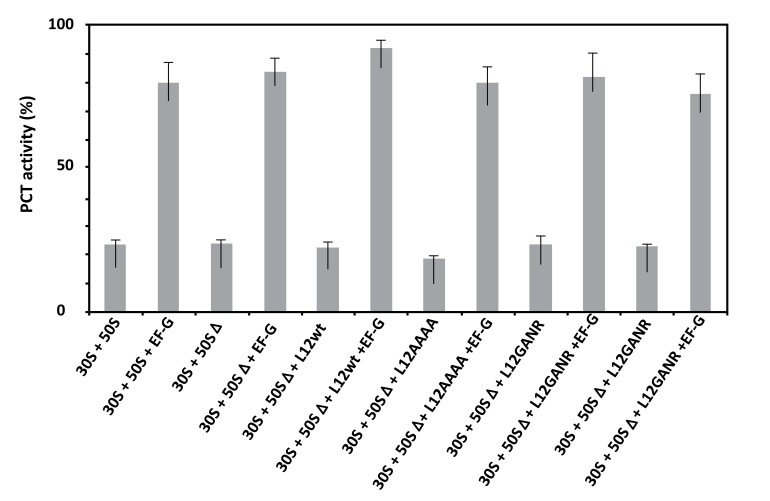
**Peptidyl transferase activity of the wild-type or mutant *E. coli* 70S ribosomes.** Peptidyl transferase activity was determined as the extent of puromycin that has reacted with the “Ac-[^3^H]Phe-tRNA.poly(U).reconstituted ribosome” complex, in the absence or the presence of the elongation factor EF-G (Materials and Methods).

Given that the activity of ribosomes in protein synthesis is composed of the peptidyl transferase reaction followed by the translocation step, the critical residue Lys-65 of rp bL12 might be involved in either of these steps at the PTC. Therefore, the *E. coli* wild-type and mutant ribosomes used in this study were tested in the peptidyl transferase reaction with respect to puromycin [33, 34]. As shown in Fig. (**5**), none of the mutations operated in the GANK motif of rp bL12 was capable of affecting the peptidyl transferase activity of the mutant ribosomes, suggesting that rp bL12 did not directly play any major role in the peptidyl transfer step of the elongation of translation. Another conclusion that can be drawn from these results is that rp bL12 did not participate in the binding of puromycin at the ribosomal A-site which represents the key step of the *in vitro* peptidyl transfer reaction. If rp bL12 was involved in the binding of puromycin at the A-site during peptidyl transfer, this reaction would not take place in its absence. In this respect, the observation that the 70S ribosomes containing the large 50SΔ subunits depleted from the original rp L7/L12 copy were found to be fully active in peptidyl transfer (Fig. **5**), while their poly(U)-dependent poly(Phe) synthesis activity was lost (Fig. **4**) needs to be clarified by a detailed description of the peptidyl transfer reaction. This reaction consisted in the formation of a covalent bond (similar to the peptidic bond) between the α-amino group of puromycin bound to the ribosomal A-site and the carbonyl carbone of Ac-[^3^H]Phe-tRNA^Phe^ positioned at the P-site, leading to the synthesis of Ac-[^3^H]Phe-puromycin (Materials and Methods). The N-terminally acetylated [^3^H]Phe-tRNA^Phe^ is supposed to preferably bind to the P-site, because it is a mimick of peptidyl-tRNA. However, one cannot exclude that a part of the Ac-[^3^H]Phe-tRNA^Phe^ binds non specifically to the A-site and competes with puromycin for binding to this site. In order to allow the N-terminally acetylated [^3^H]Phe-tRNA^Phe^ to bind in majority to the ribosomal P-site, the elongation factor EF-G was added to the incubation mixture of the peptidyl transfer reaction. This translation factor is required for the translocation of the tRNA molecule from the A-site to the P-site, or from the P-site to the E-site. Therefore, in the presence of EF-G, it is most probable that Ac-[^3^H]Phe-tRNA^Phe^ and puromycin are bound to the P-site and to the A-site, respectively. As shown in Fig. (**5**), the extent of Ac-[^3^H]Phe-puromycin synthesis was much lower in the absence of EF-G than in its presence, whatever the type of ribosome used in the peptidyl transferase assay. This result suggests that, as expected, independently of the presence of rp bL12 *in vitro*, the translation factor EF-G facilitates the binding of Ac-[^3^H]Phe-tRNA^Phe^ to the ribosomal P-site, while puromycin binds freely to the A-site.

### 3.6 Amino Acid Changes in the 62GANK65 Peptide of bL12 Result in Restricted Enzymatic Binding of Aminoacyl-tRNA to the A-site of *E. coli 70S* Ribosomes

As discussed above, the 70S ribosomes containing the large 50SΔ subunits depleted from the original rp L7/L12 copy were found to be fully active in peptidyl transfer (Fig. **5**), while their poly(U)-dependent poly(Phe) synthesis activity was lost (Fig. **4**). One obvious conclusion to be drawn from these results is that the inhibition of the poly(U)-dependent poly(Phe) synthesis activity of the mutant ribosomes is most probably caused by the enzymatic binding of aminoacyl-tRNA (aa-tRNA) to the ribosomal A-site. If this is the case, the binding of [^3^H]Phe-tRNA^Phe^ mediated by elongation factor EF-Tu to the A-site of poly(U)-programmed ribosomes would be restricted in some of the mutant ribosomes. As shown in Fig. (**6**), [^3^H]Phe-tRNA^Phe^ binding to the ribosomal A-site in the presence of EF-Tu is affected in the mutant ribosomes that exhibit a reduced poly(Phe) synthesis activity. These results suggest that the defect in the occupation of the ribosomal A-site of the mutant ribosomes is directly responsible for the inhibition of the poly(U)-dependent poly(Phe) synthesis activity. Therefore, the functional role of rp bL12 is likely to consist in the binding of the incoming aa-tRNA to the ribosomal A-site at the elongation step of translation. At this stage, one can remark that the difference between the *in vitro* synthesis of Ac-[^3^H]Phe-puromycin or poly(Phe) by the ribosome is the formation of only one peptide bond (Ac-[^3^H]Phe-puromycin synthesis) in the former and of several consecutive repeatedly connected peptide bonds (poly(Phe) synthesis) in the latter. In accordance with this observation, we have demonstrated above that the rp bL12 did not participate to the binding of puromycin at the ribosomal A-site, in sharp contrast to the binding of the incoming aa-tRNA whose entry into the A-site was shown to be strictly dependent on the interactions with rp bL12 and the elongation factor Tu (EF-Tu). In other words, rp bL12 would be dispensable for the binding of puromycin and indispensable for the binding of the incoming aa-tRNA to the ribosomal A-site. These data are likely to reflect that the number of interactions of the tRNA molecule with the *E. coli* ribosome is by far larger than that of puromycin, due to the difference in size between these molecules. In conclusion, the mechanisms of the *in vitro* peptidyl transfer reaction with the help of puromycin and of the *in vivo* naturally occurring polynucleotide-dependent polypeptide synthesis are not of the same type and do not rely on the same interactions with the ribosome. This might explain why the 70S ribosomes containing the large 50SΔ subunits depleted from the original rp L7/L12 copy are fully active in peptidyl transfer, while their poly(U)-dependent poly(Phe) synthesis activity was lost.

### 3.7 Hypothetical Overall Mechanism for the Elongation Step of Translation Catalyzed by *E. coli* 70S Ribosomes

**Fig. (7):**
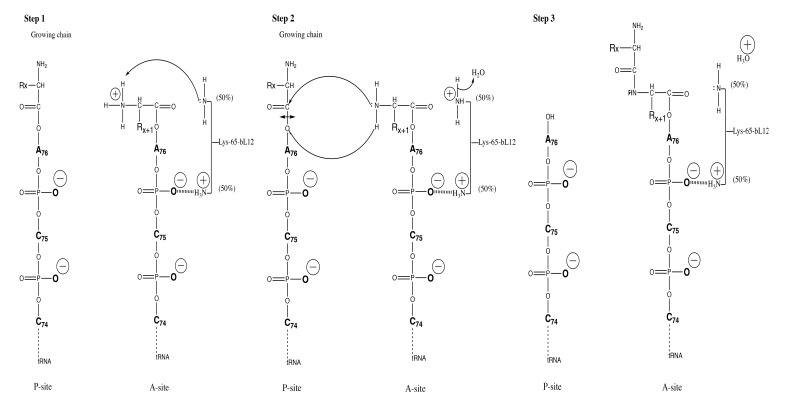
The hypothetical overall mechanism for the elongation step of translation catalyzed by *E. coli* 70S ribosomes.

The binding and correct positioning by rp bL12 of the incoming aa-tRNA at the ribosomal A-site is likely to be insured by the critical Lys-65, because the positively charged side chain of this residue is susceptible to establish ionic bonds with phosphate groups of the polyanionic tRNA molecule, especially at its single-stranded 3’-acceptor end (Fig. **7**). This situation is most probable since the protonated, positively charged εNH_3_^+^ side chain of Lys-65 can be estimated to represent about 50% of this primary amine group at neutral pH (Fig. **7**), because this residue was recently shown to exhibit a pK of 7.2 [16]. Such electrostatic interactions are consistent with the view that the ribosome provides positional catalysis in the sense that it facilitates peptide bond formation by insuring the accurate positioning of the A-site-bound aa-tRNA [35]. This orientation of the aa-tRNA is required for the spontaneous nucleophilic attack of its α-amino group on the carbonyl carbone of P-site bound peptidyl-tRNA [35]. However, it is conceivable that, in addition to the correct positioning of the tRNA substrates at the PTC, the ribosome may also catalyze the formation of peptide bond by a conventional chemical mechanism. In agreement with the latter proposition, the pH dependence of peptidyl transfer reaction catalyzed by *E. coli* ribosomes indicated the presence at the PTC of an ionizing group of the ribosome with a pK of 7.5, which could be a catalyst of peptide bond formation by the ribosome [36]. Since this group had never been identified, we propose that the catalytic residue is in fact a lysyl residue with a considerably lowered pK such as Lys-65 of bL12 (pK of 7.2) [16]. Thus, if correctly positioned at the PTC, the remaining 50% of unprotonated εNH_2_ form of the Lys-65 side chain at neutral pH would be capable of performing catalysis of the formation of peptide bond. Altogether, our results make it possible to propose the following hypothetical overall mechanism for each elongation cycle of translation: (i) the first event in the course of translation elongation consists in the binding and the correct positioning of the incoming aminoacyl-tRNA at the ribosomal A-site by rp bL12 in a complex with the elongation factor Tu (EF-Tu). As discussed above, the contribution of rp bL12 to this step would consist in electrostatic interactions between the positively charged side chain of Lys-65 and phosphate groups of the CCA-arm of the tRNA molecule. It is interesting to recall that Lys-65 is located in the large globular C-terminal domain (residues 53-120) of rp bL12 that was previously shown to interact with elongation factors including EF-Tu [17, 18, 37-39]. The second event represents the deprotonation of the α-NH_3_^+^ group of the aa-tRNA positioned at the A-site by the unprotonated εNH_2_ group of the catalytic Lys-65 side chain to generate the nucleophilic α-NH_2_ group capable of attacking the ester bond of peptidyl-tRNA. These two events constitute the first step of the mechanism (Fig. **7**, Step 1); (ii) the second step is the nucleophilic attack of the α-NH_2_ group of the aa-tRNA on the electrophilic carbonyl group of the peptidyl-tRNA in the P-site. Subsequently, peptide bond formation would stem from this nucleophilic attack, followed by the deacylation of the P-site tRNA, as well as the protonation of its 3’-end (Fig. **7**, Step 2); (iii) finally, the third step would permit the restoration of the nucleophilic ε-NH_2_ group of Lys-65 following donation of a proton by ε-NH_3_^+^ to a water molecule. This last step makes it possible to initiate a next elongation cycle by abstracting a proton from the α-NH_3_^+^ group of the next incoming aa-tRNA (Fig. **7**, Step 3). It should be noted that the mechanism of peptide bond formation stemming from this study is strongly supported by the following previously reported data: (i) direct visualization of A-, P-, and E-sites transfer RNAs in the *E. coli* ribosome by cryo-EM studies had revealed that the A-site is localized close to the L7/L12 stalk [40], in accordance with the participation of this protein to the binding of the incoming aminoacyl-tRNA to the ribosomal A-site; (ii) affinity labeling of Lys-65 of rp bL12 with a reactive tRNA analogue *in situ* on the *E. coli* 70S ribosomes was shown to provoke the loss of activity [16], suggesting that this residue plays a functional role critical for ribosomal activity. Altogether, our findings challenge the ribozyme-catalyzed peptidyl transfer mechanism and, at the same time, provide new insights into the mechanism of peptide bond formation by the ribosome.

## CONCLUSION

In this report, we have used mutational analysis of the GGQ-like 62GANK65 motif of rp bL12 to demonstrate that Lys-65 of this motif is critical for the activity of *E. coli* 70S ribosomes. We show that, with the exceptions of the K65R or K65H substitutions, all mutations operated at the position of Lys-65 of the GANK motif of bL12 led to a severe impairment in the poly(U)-dependent poly(Phe) synthesis activity of the ribosomes containing the mutant proteins. This result suggests that the only requirement of the reaction catalyzed or facilitated by rp bL12 on the ribosome is the positive charge of the side chain of the amino acid residue at the position of Lys-65. We also demonstrate that the Lys-65 residue did not play any role in the peptidyl transferase activity, but rather assists the enzymatic binding of aminoacyl-tRNA to the ribosomal A-site. The fraction of protonated, positively charged εNH_3_^+^ side chain of Lys-65 is likely to participate to the binding of the incoming aminoacyl-tRNA through ionic bonds with phosphate groups of the polyanionic tRNA molecule, especially at its single-stranded 3’-acceptor end. We propose that, in addition, the fraction of unprotonated εNH_2_ form of the Lys-65 side chain performs catalysis of the formation of peptide bond by withdrawing a proton from the α-NH_3_^+^ group of the aa-tRNA positioned at the A-site to generate a nucleophilic α-NH_2_ group. Subsequently, peptide bond formation would stem from the nucleophilic attack of this α-NH_2_ group on the electrophilic carbonyl group of the peptidyl-tRNA.

## Figures and Tables

**Fig. (1A):**
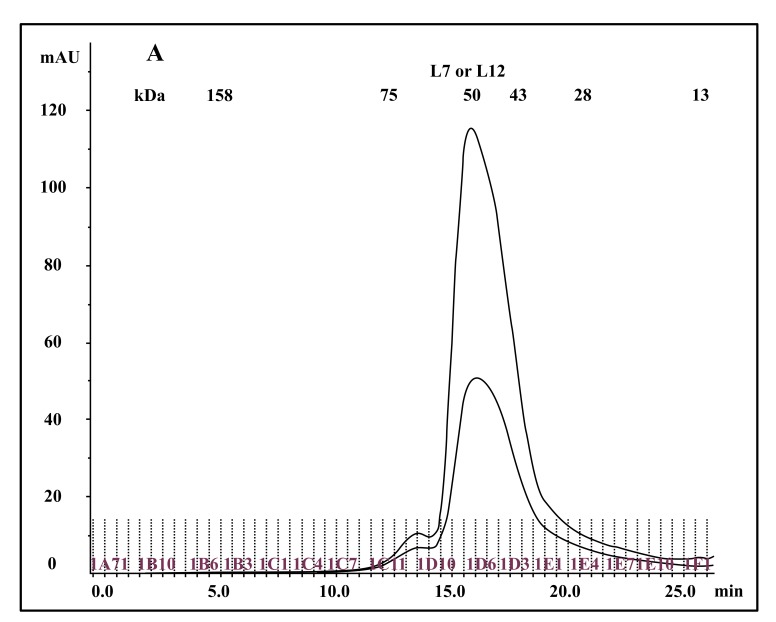
**Analysis of the recombinant bL12 protein on a Superdex 75 column.** (**A**) The column was equilibrated in 50 mM sodium phosphate buffer (pH 7.5) containing 150 mM NaCl. The flow rate was 0.4 ml/min. Elution was monitored by the absorbance at 280 nm (upper peak) and 260 nm (lower peak). The column was calibrated with protein markers of molecular weights 13, 28, 43, 75 and 158 kDa. Analysis on the column of rp (L7/L12)_2_ with two free NH_2_-terminus L12 chains and two NH_2_-terminally acetylated L7 chains, as well as of the fully acetylated (L7)_4_ form gave rise to superimposable elution profiles. Fractions of 0.4 ml were collected. (**B**) Analysis in 10% SDS-PAGE of the fractions eluted from the Superdex 75 column. Fractions 15-20 named L12 represent the rp (L7/L12)_2_ with two free NH_2_-terminus L12 chains and two NH_2_-terminally acetylated L7 chains, while fractions 15’-20’ named L7 represent the fully acetylated (L7)_4_ form. The latter rp (L7)_4_ was obtained by incubating the (L7/L12)_2_ form with two free NH_2_-terminus L12 chains and two NH_2_-terminally acetylated L7 chains in the presence of purified RIML acetyl-transferase and of N-acetyl coenzyme A (N-Ac-CoA). For details, see Materials and Methods. The SDS-PAGE gels were colored with coomassie blue.

**Fig. (1B):**
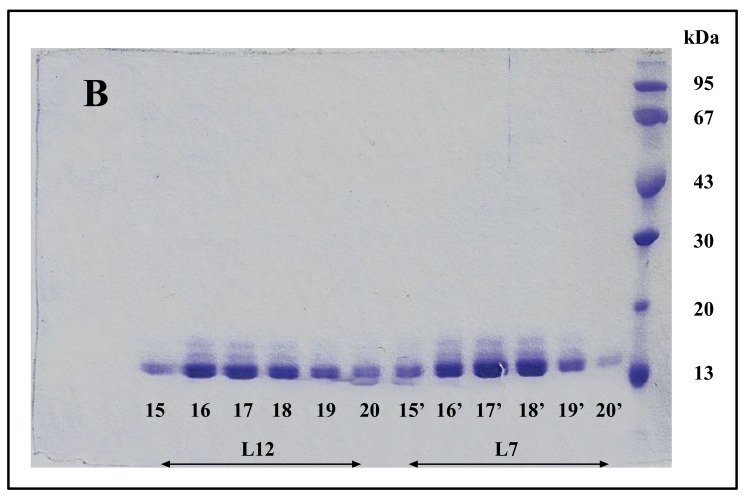
**Analysis of the recombinant bL12 protein on a Superdex 75 column.** (**A**) The column was equilibrated in 50 mM sodium phosphate buffer (pH 7.5) containing 150 mM NaCl. The flow rate was 0.4 ml/min. Elution was monitored by the absorbance at 280 nm (upper peak) and 260 nm (lower peak). The column was calibrated with protein markers of molecular weights 13, 28, 43, 75 and 158 kDa. Analysis on the column of rp (L7/L12)_2_ with two free NH_2_-terminus L12 chains and two NH_2_-terminally acetylated L7 chains, as well as of the fully acetylated (L7)_4_ form gave rise to superimposable elution profiles. Fractions of 0.4 ml were collected. (**B**) Analysis in 10% SDS-PAGE of the fractions eluted from the Superdex 75 column. Fractions 15-20 named L12 represent the rp (L7/L12)_2_ with two free NH_2_-terminus L12 chains and two NH_2_-terminally acetylated L7 chains, while fractions 15’-20’ named L7 represent the fully acetylated (L7)_4_ form. The latter rp (L7)_4_ was obtained by incubating the (L7/L12)_2_ form with two free NH_2_-terminus L12 chains and two NH_2_-terminally acetylated L7 chains in the presence of purified RIML acetyl-transferase and of N-acetyl coenzyme A (N-Ac-CoA). For details, see Materials and Methods. The SDS-PAGE gels were colored with coomassie blue.

**Fig. (2A):**
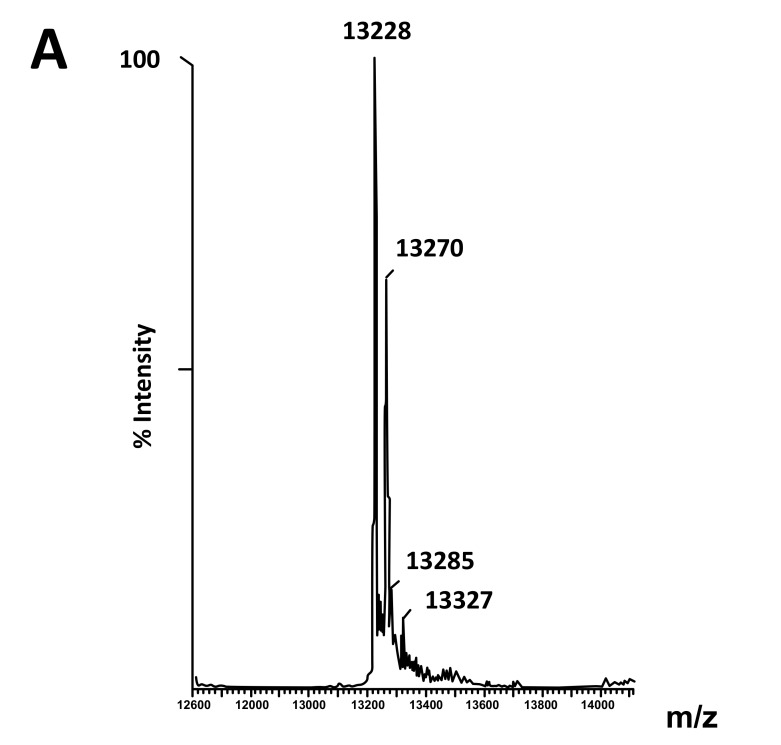
**Analysis by MALDI mass spectrometry of rps (L7/L12)_2_ or (L7)_4_.** (**A**): MALDI mass spectrum of the control recombinant rp (L7/L12)_2_. The molecular weights measured are 13228 Da (L12 chain) and 13270 Da (acetylated L7 chain). The 42 Da difference between the two masses represents the acetyl group. The peaks at 13285 Da and 13327 Da correspond respectively to the acetylated L7 chain (MW 13270 Da) modified with a methyl group (MW 15 Da) or with both a methyl group and a supplementary acetyl group (total mass increase of 57 Da). (**B**): MALDI mass spectrum of the fully acetylated rp (L7)_4_. The peak of mass 13274 Da represents the acetylated L7 chain. The peak of mass 13499 Da showing a difference of 225 Da represents the L7 chain attached to additional acetyl and methyl groups. Both (L7/L12)_2_ and (L7)_4_ forms were eluted from the Superdex column, and the fractions of interest were pooled and evaporated prior to MALDI mass spectrometric analyses. For details, see Materials and Methods.

**Fig. (2B):**
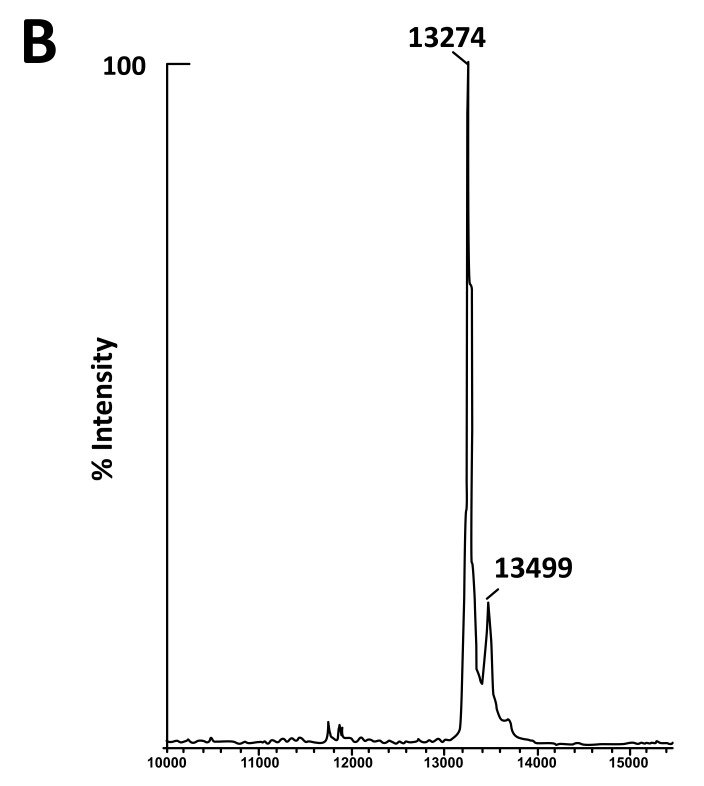
**Analysis by MALDI mass spectrometry of rps (L7/L12)_2_ or (L7)_4_.** (**A**): MALDI mass spectrum of the control recombinant rp (L7/L12)_2_. The molecular weights measured are 13228 Da (L12 chain) and 13270 Da (acetylated L7 chain). The 42 Da difference between the two masses represents the acetyl group. The peaks at 13285 Da and 13327 Da correspond respectively to the acetylated L7 chain (MW 13270 Da) modified with a methyl group (MW 15 Da) or with both a methyl group and a supplementary acetyl group (total mass increase of 57 Da). (**B**): MALDI mass spectrum of the fully acetylated rp (L7)_4_. The peak of mass 13274 Da represents the acetylated L7 chain. The peak of mass 13499 Da showing a difference of 225 Da represents the L7 chain attached to additional acetyl and methyl groups. Both (L7/L12)_2_ and (L7)_4_ forms were eluted from the Superdex column, and the fractions of interest were pooled and evaporated prior to MALDI mass spectrometric analyses. For details, see Materials and Methods.

**Fig. (6):**
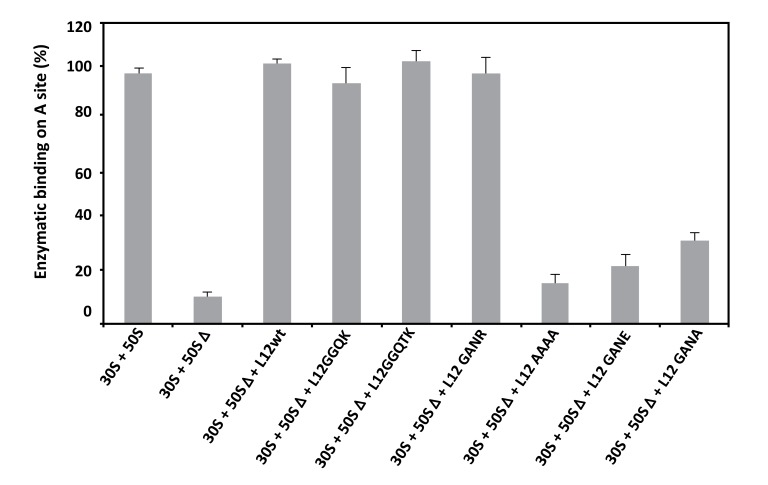
**Enzymatic binding of [^3^H]Phe-tRNA^Phe^ to the ribosomal A-site of poly(U)-programmed ribosomes reconstituted with wild-type or mutant rps bL12.** The 100% activity corresponds to the enzymatic binding of [^3^H]Phe-tRNA^Phe^ to the wild-type ribosomes obtained by the combination of equimolar amounts of the *E. coli* small (30S) and large (50S) ribosomal subunits in the presence of EF-Tu•GTP. Non enzymatic binding was deduced (Materials and Methods).

## LIST OF ABBREVIATIONS

PTC: = Peptidyl transferase center
rp: = Ribosomal protein
bL12 (or RpbL12): = The new name of eubacterial large subunit ribosomal protein L12 with a free NH_2_ terminus, or its NH_2_-terminally acetylated form L7 (also called bL7)
eL42: = eukaryal or archaeal large subunit ribosomal protein L42 (formerly L42A or L42AB in yeast or L36a in human, or L44e in archaea)
uL10: = The large subunit ribosomal protein L10 present in the three major kingdoms of life
Ac-CoA: = Acetyl-coenzyme A.
